# Supplementary material for: Primordial germ cells adjust their protrusion type while migrating in different tissue contexts in vivo
Source: Development. 2023 Jan 16;150(2):dev200603. doi: 10.1242/dev.200603 (PMC10110502; doi:10.1242/dev.200603)
Supplement: Supplementary file 1 [file develop-150-200603_review_history.pdf]

Primordial germ cells adjust their protrusion type while migrating in different tissue contexts *in vivo*

Lukasz Truszkowski, Dilek Batur, Hongyan Long, Katsiaryna Tarbashevich, Bart E. Vos, Britta Trappmann and Erez Raz
DOI: 10.1242/dev.200603

Editor: Thomas Lecuit

Review timeline

Original submission:	31 January 2022
Editorial decision:	21 April 2022
First revision received:	27 October 2022
Accepted:	29 November 2022

Original submission

First decision letter

MS ID#: DEVELOP/2022/200603

MS TITLE: Primordial germ cells adjust their protrusion type while migrating in different tissue contexts in vivo

AUTHORS: Lukasz Truszkowski, Dilek Batur, Hongyan Long, Bart E Vos, Britta Trappmann, and Erez Raz

I apologize for the long delay before coming back to you. One of the reviewers fell ill during the review process but has now recovered. I have now received all the referees' reports on the above manuscript, and have reached a decision. The referees' comments are appended below, or you can access them online: please go to BenchPress and click on the 'Manuscripts with Decisions' queue in the Author Area.

As you will see, the referees express strong interest in your work, but have some significant criticisms and recommend a substantial revision of your manuscript before we can consider publication. If you are able to revise the manuscript along the lines suggested, which may involve further experiments, I will be happy receive a revised version of the manuscript. Your revised paper will be re-reviewed by one or more of the original referees, and acceptance of your manuscript will depend on your addressing satisfactorily the reviewers' major concerns. Please also note that Development will normally permit only one round of major revision. If it would be helpful, you are welcome to contact us to discuss your revision in greater detail. Please send us a point-by-point response indicating your plans for addressing the referee’s comments, and we will look over this and provide further guidance.

Please attend to all of the reviewers' comments and ensure that you clearly highlight all changes made in the revised manuscript. Please avoid using 'Tracked changes' in Word files as these are lost in PDF conversion. I should be grateful if you would also provide a point-by-point response detailing how you have dealt with the points raised by the reviewers in the 'Response to Reviewers' box. If you do not agree with any of their criticisms or suggestions please explain clearly why this is so.

© 2023. Published by The Company of Biologists under the terms of the Creative Commons Attribution License (https://creativecommons.org/licenses/by/4.0/).

Reviewer 1

Advance summary and potential significance to field

The manuscript by Truszkowski and colleagues addresses the impact of surrounding tissues on the motile behavior of primordial germ cells (PGCs). Building on their previous work, they show that PGCs alter their polarized protrusion behavior when interacting with dextran gels of different stiffness and go on to show differences in protrusion size and dynamics when migrating through ectoderm and mesodermal tissues, with the authors concluding that this is due to differences in the mechanics of surrounding tissues. They further characterize this differential response using live reporters and propose a model that couples extrinsic inputs to the self-organizing polarization mechanism that they have recently described.

This is a well-written manuscript that addresses the important and timely question of how extrinsic changes can influence cell autonomous behaviors. This reviewer appreciates this focus and the powerful model system.

Comments for the author

Some of the experiments in the current version are insufficiently characterized or controlled, to the level that is not yet clear that these are changing the system in the way proposed, so the authors should provide further experimental support for these claims. A related issue that the study offers a narrow interpretation for how surrounding tissues influence migrating cell behavior, when previous work from the same group appears to offer alternative explanations (these alternatives are also not covered in the discussion). Combined, these shortcomings give the impression of a study that is too preliminary in investigation and too narrow in interpretation to yet be useful for the Development readership. I will briefly offer suggestions as how best to address these issues.

1. The relationship between protrusion frequency/size and polarity

Previous work by the Raz group has shown that PGCs change their protrusive behavior when interacting with surrounding tissues, with the behavioural readout usually being a change or loss of protrusion polarity.
Presumably polarity changes are obvious because the cells in these contexts 'collide' in an asymmetric manner with other tissues. Truszkowski et al take this further by monitoring the interaction of PGCs with dextran gels of different mechanical properties. This reviewer appreciates these experiments as they bring the benefits of in vitro approaches to a more physiological in vivo context. They report that cells that PGCs colliding with stiffer gels are more likely to change or lose their polarity than those contacting with softer gels. This reaction looks very similar to the reaction of PGCs when contacting E-cadherin deficient cell clones, which also lose or change their polarity, suggesting that the reaction of PGCs to stiffer gels and E-cadherin deficient tissues is via the same mechanism (Grimaldi et al 2020).

One obvious question is how do the data on the dextran gels relate to the behaviour of cells migrating in ectoderm-only or mesoderm-only embryos? The problem here is that the reaction of cells to different tissue environments is measured in terms of protrusion frequency and size, not polarity changes (I assume that the polarity differences were less obvious due to the fact that the cells migrate through a more uniform environment?). This disconnect in how the data are measured makes it difficult to integrate these findings - is the reaction to the gels and different tissue environments due to the same underlying mechanism?

Recommendation: The same readout of behavior should be used in both sets of experiments to make them more directly comparable. Alternatively, the authors can provide another way to integrate these findings.

2. Characterization of ‘single-germ-layer’ embryos

This reviewer found the experiments found the experiments where guidance receptor deficient PGCs were transplanted of into ‘single-germ-layer’ embryos ingenious (and I’m confident that other readers will enjoy these too). The authors reach the conclusion that the different behavior of PGCs

© 2023. Published by The Company of Biologists under the terms of the Creative Commons Attribution License (https://creativecommons.org/licenses/by/4.0/).

is due to the cortical mechanical differences between ectodermal and mesodermal cells already described by Krieg et al (2008).
Unfortunately, their single attempt to support this idea, by reducing the higher cortical tension in ectoderm cells by injecting DN-ROCK, only led to a reduction of bleb frequency but not size, a somewhat counterintuitive finding. More importantly, there are other possible explanations for the differences between tissues. For example, it's likely that embryos comprised entirely of ectoderm are organized differently from embryos comprised of mesoderm, in terms of cell numbers and densities, not to mention predictable biochemical differences (see point 3).

Recommendation: The single-germ-layer embryos should be better characterized to support the proposal that the described effects are due specifically to cortical tension differences. How are cells organized in each case, in terms of numbers and packing? This could be addressed by simple labelling.Are there differences in the size/number of gaps between cells that could explain the observed difference in blebbing? I could not find this information in the cited Krieg et al (2008) paper, which mainly used this elegant trick to investigate cell sorting in explants. This could be addressed by labelling the interstitial spaces by injecting fluorescent transfers or expressing secreted FPs.

For a paper that focusses on the impact of tissue environment there is little investigation into the respective tissue environments – almost all of the data show labelled PGCs on a black background.

3. Excluding (or integrating) the previously described role for cadherins in regulating PGC blebbing

Previous work by the Raz lab has shown that differences in e-cadherin activity in surrounding tissues can influence PGC blebbing (albeit measured as a change in polarity). Given that ectoderm-only and mesoderm-only embryos will show differences in e-cad activity, this reviewer is surprised that this alternative interpretation was not explored here, or even mentioned in the discussion. While the bulk expression of e-cad may not vary detectably, due to the known maternal expression, it is highly likely that these germ layers will differ in e-cad activity, due to differences in stability, presentation etc. Moreover, it is predictable that mesoderm-only embryos will have n-cad activity which could also have an effect via a cadherin-based polarization mechanism.

Recommendation: The authors should rule out that the differences in PGC behavior in different tissues are not due to the e-cadherin based polarisation mechanism that they've previously described. If there are already compelling arguments against these should be covered in the discussion (the Grimaldi paper is currently cited as a source of methods but the biological findings of this work are important to discuss here).

Minor

4. Figure 4 investigates how differences in PGC 'self-organized polarity' could explain the described differences in bleb frequency. Using live reporters, they show that both RhoA activity is increased and erzin localization is expanded towards the cell front in ectodermal environments, making these cells less likely to bleb. The model they present - where a 'moderate', not high, increase in RhoA activity increases cortical coupling and thus prevents blebs - is a nice extension of that first presented in their Olguin-Olguin paper.
However, here the new idea is not tested experimentally and is thus less convincing than the previous study.

Reviewer 2

Advance summary and potential significance to field

Truszkowski et al. explore how migrating Zebrafish primordial germ cells (PGCs) acutely respond to and adapt to cellular environments with different physical properties. The motivations for this study are twofold- (1) Zebrafish PGCs are born in distinct locations and may need to migrate within different cellular contexts with potentially different properties, and (2) previous work in the Raz lab indicated that PGC motility is obstructed by different barriers during developmental migration, such as the gut and notochord. To decouple whether the acute response to barriers (Loss of

© 2023. Published by The Company of Biologists under the terms of the Creative Commons Attribution License (https://creativecommons.org/licenses/by/4.0/).

polarity) is due to mechano-sensing vs signaling, they inject hydrogels into the embryo and observe how non-directionally migrating PGCs respond. These experiments are followed up by observing PGCs in ectopically transformed embryos that are all ectoderm or mesoderm with the ectoderm previously shown to have higher cortical tension. PGCs can surprisingly migrate with a similar speed and persistence in either cellular environment due to an increase in actin-based pseudopod motility at the expense of bleb-based motility in the ectoderm. The adaptive mechanism put forth is that PGCs increase RhoA activity globally and distribute ezrin more evenly across the membrane as to restrict bleb formation. The conclusions and experiments are generally sound but require further clarification with additional data/revisions to the text.

Comments for the author

Comments-
1. Line 93- The authors state that the hydrogels utilized (Fig. S1) have physical properties measured as young's modulus which are within the physiological range that a PGC might encounter. However, there is no discussion of the associated physical properties (i.e. young's modulus measurement) of the embryonic cells that PGCs normally migrate within. For the previously described barriers that cause a repolarization of PGC migration (gut, notochord), is the stiffer gel within the range of these structures?

2. In the hydrogel experiments, the PGCs, at least from the included movies and still images, primarily use actin-rich protrusions rather than the blebs one typically associates with these PGCs. This begs the question how do PGC blebs interact with these hydrogels and if the apparent stiffness sensing mechanism is dependent upon a branched actin network present in the pseudopod.

3. Are PGC migration speed and track straightness similar when migrating within control and dominant negative ROCK expressing ectoderm? If one assumes the cell surface molecule repertoire remains similar this data could provide evidence that PGCs are adapting to the physical properties of the ectoderm rather than responding to surface molecules on the ectoderm vs mesoderm. The ectoderm and mesoderm express different levels of E-cadherin and this group has noted changes in PGCs migration in response to environmental cadherin levels. Can changes in E-cadherin levels on PGCs or environment alter morphology?

4. Along these lines, it would be interesting to see if bleb size and frequency also decrease when PGCs are migrating within mesoderm with increased cortical tension through expression of constitutively active ROCK etc. This would show that this adaptive mechanism is more general and not specific to ectoderm.

5. Although identifying the causative upstream mechanism in which PGCs adapt to increased compression from the cellular environment is beyond the scope of this work, it would be interesting to see if PGCs which are "locked" into a particular migration mode fail to adapt when migrating within all ectoderm vs mesoderm.
For example, in previous work from this group, PGCs overexpressing MLCK extend elongated protrusions and are still able to migrate.

6. Please include representative FRET images for RhoA and Rac1 in Fig 4.

<u>Reviewer 3</u>

Advance summary and potential significance to field

In this paper, Truszkowski et al investigate zebrafish primordial germ cells (PGCs) migrating within different embryonic germ layers, and find that PGC migratory behavior is different between ectoderm and mesoderm environments. They show that PGCs in ectoderm produce smaller and less frequent blebs and more actin-rich protrusions than cells within mesodermal tissue. They also suggest a mechanism: PGCs within ectoderm tissue display elevated RhoA activity, which the authors propose leads to increased ERM activity and thus reduced blebbing. Finally, the authors propose that differences in tissue mechanics between ectoderm and mesoderm cause the observed differences in PGC behavior.

© 2023. Published by The Company of Biologists under the terms of the Creative Commons Attribution License (https://creativecommons.org/licenses/by/4.0/).

The work is novel and potentially interesting. PGCs are a rare example of cells that migrate through changing environments in vivo in physiological conditions. They are thus an excellent system to study how the environment affects cell migration, and the findings presented here might have important impact in other systems where migrating cell face environmental changes, e.g. during cancer dissemination. However, some of the key conclusions are not, at this point, supported by the experiments, and several other points need to be addressed either through experiments or through clarifications.

Comments for the author

My main concern lies with the interpretation proposed by the authors that the differences in migratory behavior they observe are due to differences in mechanics between ectoderm and mesoderm. This conclusion is, at present, poorly supported by the data, and the discussion of mechanics is overall very confusing. Specifically:

1. The authors first test the effects of mechanics by introducing hydrogels of different stiffnesses into an embryo – Figure S1. However, the data presented in this figure are not fully convincing. Given the low numbers, the conclusion relies on a few cells displaying a “polarity loss” rather than “no change” upon contacting stiffer gels. But is 5 or 6 minutes long enough a movie for this analysis? Have the authors tried observing for a longer time? For instance, in the videos presented the cells approach the hydrogels at quite different angles, could it be that “no change” turns into “turn” or “polarity loss” if observed for longer? More fundamentally, the rationale of this experiment within the context of the paper is unclear: how does hydrogel stiffness relate to cortical tension of neighboring cells? Stiffness and tension are very different physical properties (see more on this below).

2. A directly related question is how do the authors propose that the cells achieve mechanosensing, and what do they actually sense? To my knowledge, current understanding of substrate stiffness sensing is that it relies on focal adhesions. Do PGCs form focal adhesions? And if not, how do the authors propose they could sense hydrogel stiffness? If this is a focal adhesion independent stiffness sensing mechanism, that would be a very exciting finding that should be explicitly discussed, but there is at this point insufficient evidence that stiffness sensing takes place at all in this system.

3. For the cells migrating in mesoderm and ectoderm: the authors write several times that these two tissues exhibit different biophysical properties, referring to Krieg’s 2008 paper. However, to my knowledge that paper measured the cortical tension of individual cells isolated from ectoderm- and mesoderm-converted embryos. It is not clear at all how the mechanical properties of single isolated cells translates to the mechanical properties of the tissue. The authors refer to tissue tension but also sometimes to cell tension within the tissue. Tissue tension is likely more determined by the ECM than by the mechanics of individual cells. The authors need to clarify that, as I presume, they mean to refer to the mechanics of the individual cells, not of the tissue (or provide some references on actual tissue mechanics).

4. The authors also occasionally use the word stiffness, apparently as a synonym to tension (e.g. l . 140).
Stiffness is a very different physical property to cortical tension, do the authors actually mean stiffness in these instances? It is plausible that cortical tension could be sensed in a similar manner to stiffness, however in principle, tension does not directly correlate with stiffness. Assuming they mean tension, as this is what has been measured in Krieg et al 2008, it would be interesting to speculate how they propose cells might sense cortical tension. Some previous work by the authors reported that PGCs form cadherin bonds to neighboring cells, is this where they propose cortex tension would be sensed?

5. The evidence that the differences in PGC behavior are actually due to differences in cell mechanics in the different tissues is rather minimal, the conclusion appears to rely on the observed increase in blebbing frequency (but not size) in dn rock ectoderm hosts. This is very correlative and the authors need to either substantiate the experimental support for their conclusion (e.g. by investigating how the mechanosensing is achieved), or strongly tone down their conclusions.

© 2023. Published by The Company of Biologists under the terms of the Creative Commons Attribution License (https://creativecommons.org/licenses/by/4.0/).

Overall, unless further data more clearly supporting a role for mechanics can be provided, I would suggest considerably toning down the statements that the differences in behavior observed are due to differences in mechanics and either removing the hydrogel experiment or moving it to the end of the paper, as a speculation point rather than a starting point.

Minor comments:

1. In the introduction, the authors present PGCs as “an accessible in vivo model” for how cell migration adapts to the environment. Do PGCs actually move between ectoderm to mesoderm in vivo (the authors suggest they do, but only rather late in the paper, l. 160, maybe this should be discussed earlier / more extensively?), and if so, is it possible to assess if they display the same changes in protrusion formation?

2. “cells can alter their protrusion type or migration mode when located in different 2D and 3D environments” (72-73): this should reference Liu’s 2015 paper (which is cited later in the manuscript), which reports an extensive study of the behavior of numerous cell types in confinement.

3. In the mutant embryo, the authors observe PGCs within all 3 germ layers: ectoderm, mesoderm, and endoderm. Yet there is no further discussion of PGCs in the endoderm. Could the authors clarify why they focused on the ectoderm and mesoderm for the rest of the study?

4. In the single germ layer embryos, are cells integrated within the tissue or is it possible they lie on the tissue boundary (i.e. between tissue and yolk)? A figure in the style of S2A for the converted embryos would be useful.

5. “These results [that PGCs in ectoderm form fewer/smaller blebs] were further supported by analyzing blebbing in wild-type PGCs transplanted into either ectoderm-converted or control embryos”. Why was this not done in the mesoderm-converted embryo also?

6. From the methods, it appears that many of the quantifications were done manually (e.g. Figure 2 bleb frequency and size, Figure S1 behavior quantifications). This is not a problem per se, however, given the small numbers, such manual quantifications should be done double-blinded. Was this the case?

7. Could the authors show a 3-dimensional rendering of the protrusions shown in Figure 3B? Are these flat lamellipodia-like protrusions or 3D shaped? And if they are not flat, is it entirely clear they are not some form of blebbing? Movie 4 does, at first glance, appear to show some bleb-like activity, at least from the 2D projection shown.

8. Examples of FRET images quantified in Figure 4A-C should be provided.

9. Figure 4C shows a much wider distribution in the RhoA front/back ratio of PGCs in ectoderm compared to mesoderm. Is this meaningful? Also, could the authors provide the actual p-values, rather than “ns”?

10. Figure S2A is a projection of a 3D stack, and therefore does show magenta specks within the PGC (especially center – mesoderm – image). Since the purpose of this figure is to demonstrate the mutual exclusivity of germ layer markers and PGC markers, is there another way to represent this that would be clearer?

© 2023. Published by The Company of Biologists under the terms of the Creative Commons Attribution License (https://creativecommons.org/licenses/by/4.0/).

First revision

Author response to reviewers' comments

We thank the reviewers very much for the constructive comments and suggestions, which we have addressed in the current, revised version of the manuscript.
Below we provide our responses to the specific points and describe the material added to the paper, typed within the comments of the reviewers. We hope the referees find this version of the manuscript suitable for publication in the Journal as a research report.

Reviewer 1 Advance Summary and Potential Significance to Field: The manuscript by Truszkowski and colleagues addresses the impact of surrounding tissues on the motile behavior of primordial germ cells (PGCs). Building on their previous work, they show that PGCs alter their polarized protrusion behavior when interacting with dextran gels of different stiffness and go on to show differences in protrusion size and dynamics when migrating through ectoderm and mesodermal tissues, with the authors concluding that this is due to differences in the mechanics of surrounding tissues. They further characterize this differential response using live reporters and propose a model that couples extrinsic inputs to the self-organizing polarization mechanism that they have recently described.
This is a well-written manuscript that addresses the important and timely question of how extrinsic changes can influence cell autonomous behaviors. This reviewer appreciates this focus and the powerful model system.

We thank the reviewer for the positive view of the work and for the helpful comments and suggestions.

Reviewer 1 Comments for the Author:
Some of the experiments in the current version are insufficiently characterized or controlled, to the level that is not yet clear that these are changing the system in the way proposed, so the authors should provide further experimental support for these claims. A related issue that the study offers a narrow interpretation for how surrounding tissues influence migrating cell behavior, when previous work from the same group appears to offer alternative explanations (these alternatives are also not covered in the discussion). Combined, these shortcomings give the impression of a study that is too preliminary in investigation and too narrow in interpretation to yet be useful for the Development readership. I will briefly offer suggestions as how best to address these issues.

We hope that with the corrections, clarifications and additional experiments we conducted, the revised version of the research report is useful for the readers. Below, we provide responses to the specific issues, and suggestions.

1. **The relationship between protrusion frequency/size and polarity**

Previous work by the Raz group has shown that PGCs change their protrusive behavior when interacting with surrounding tissues, with the behavioural readout usually being a change or loss of protrusion polarity. Presumably polarity changes are obvious because the cells in these contexts 'collide' in an asymmetric manner with other tissues. Truszkowski et al take this further by monitoring the interaction of PGCs with dextran gels of different mechanical properties. This reviewer appreciates these experiments as they bring the benefits of in vitro approaches to a more physiological in vivo context. They report that cells that PGCs colliding with stiffer gels are more likely to change or lose their polarity than those contacting with softer gels. This reaction looks very similar to the reaction of PGCs when contacting E-cadherin deficient cell clones, which also lose or change their polarity, suggesting that the reaction of PGCs to stiffer gels and E-cadherin deficient tissues is via the same mechanism (Grimaldi et al 2020).

The results reported in the Grimaldi et al paper suggest that PGC turning away from domains of low E-cadherin levels is a manifestation of an increase in retrograde flow at the side of contact. According to the proposed model, the increased flow reduces actin filament concentration and thereby the likelihood of bleb/front formation at this side of the cell, resulting in a turn. In the

© 2023. Published by The Company of Biologists under the terms of the Creative Commons Attribution License (https://creativecommons.org/licenses/by/4.0/).

case of the gels, as the referee points out, a similar mechanism is likely to contribute, or be responsible for the turning behavior of the cells. However, two considerations led us to conduct the more challenging gel experiments. First, these experiments rule out the formal possibility that in the case of the "asymmetric E-cadherin interaction", secondary signals provided by the live tissue (or lack of them) influence the behavior of the PGCs (e.g. the group of E-cadherin depleted cells provide a repulsive signal). The gel- based experiments allowed us to unequivocally determine the effect of purely physical signals.
Second, they allowed us to determine if cells can sense and respond to differences in stiffness. We now explain these points in the main text of the revised manuscript (lines 203-217).

One obvious question is how do the data on the dextran gels relate to the behaviour of cells migrating in ectoderm-only or mesoderm-only embryos? The problem here is that the reaction of cells to different tissue environments is measured in terms of protrusion frequency and size, not polarity changes (I assume that the polarity differences were less obvious due to the fact that the cells migrate through a more uniform environment?). This disconnect in how the data are measured makes it difficult to integrate these findings - is the reaction to the gels and different tissue environments due to the same underlying mechanism?

Recommendation: The same readout of behavior should be used in both sets of experiments to make them more directly comparable. Alternatively, the authors can provide another way to integrate these findings.

We did not employ the same readout for gel experiments and experiments in converted germ layers, due to the difference in the time scale and nature of the interaction with the gels (short and local) and with cells of the two germ layers (long and broad). Accordingly, the response of PGCs is rapid in case of gels (polarity change), and continuous in germ layers (protrusion type change). Nevertheless, we consider the finding that PGCs can sense and respond to stiffness (the gel experiments) relevant for the migration in the different environments that can differ in this parameter.
As suggested, in the current version, we integrate the findings obtained in the gel experiments differently and present them towards the end of the report. We state there that results of the gel experiments show that in principle, the reaction of the PGCs to the two germ layers could involve sensing of stiffness (lines 203-222).

2. Characterization of 'single-germ-layer' embryos

This reviewer found the experiments found the experiments where guidance receptor deficient PGCs were transplanted of into 'single-germ-layer' embryos ingenious (and I'm confident that other readers will enjoy these too). The authors reach the conclusion that the different behavior of PGCs is due to the cortical mechanical differences between ectodermal and mesodermal cells already described by Krieg et al (2008). Unfortunately, their single attempt to support this idea, by reducing the higher cortical tension in ectoderm cells by injecting DN-ROCK, only led to a reduction of bleb frequency but not size, a somewhat counterintuitive finding. More importantly, there are other possible explanations for the differences between tissues. For example, it's likely that embryos comprised entirely of ectoderm are organized differently from embryos comprised of mesoderm, in terms of cell numbers and densities, not to mention predictable biochemical differences (see point 3).

Recommendation: The single-germ-layer embryos should be better characterized to support the proposal that the described effects are due specifically to cortical tension differences. How are cells organized in each case, in terms of numbers and packing? This could be addressed by simple labelling. Are there differences in the size/number of gaps between cells that could explain the observed difference in blebbing? I could not find this information in the cited Krieg et al (2008) paper, which mainly used this elegant trick to investigate cell sorting in explants.

This could be addressed by labelling the interstitial spaces by injecting fluorescent transfers or expressing secreted FPs.

© 2023. Published by The Company of Biologists under the terms of the Creative Commons Attribution License (https://creativecommons.org/licenses/by/4.0/).

As suggested by the reviewer, to characterize the cell density of converted embryos, we determined the distance between the nuclei in 3D (new Fig. S3, lines 97-99 in main text). The results of this experiment show that as judged by this criterion, there is no difference in cell packing. As suggested, we also labelled the interstitial space by injection of labelled dextran. However as can be observed also in Krens et al., 2017, even in a non-manipulated embryo the size and intensity of the signal are very variable (see also Figure R1). Due to the large variability in signal level and gap sizes, we did not think it is appropriate to use this method for evaluating the packing, and mention in the text only the nuclei-based measurements.

Concerning the finding that cortical tension affects the frequency and not the bleb size, our view as presented in lines 107-111 is the following: We confirmed the findings by transplanting wild-type cells into embryos in which the cortical tension was increased (obtaining less blebs, new Fig 2G-I), or decreases (obtaining more blebs, Fig 2D-F) . The changes in cortical tension affected only frequency of the blebs, but not their size. As discussed in the manuscript, these findings are consistent with the idea that cortical tension in neighboring cells inhibit the initiation of blebs, but does not affect their expansion. We thus conclude that the degree of bleb expansion is controlled by properties of the environment different from cortical tension. To explain this better, we changed the text in the abstract to make it clear that cortex tension is just one parameter that affect the behavior (lines 34-35) and similarly in the main text (lines 67-68 and 107-111).

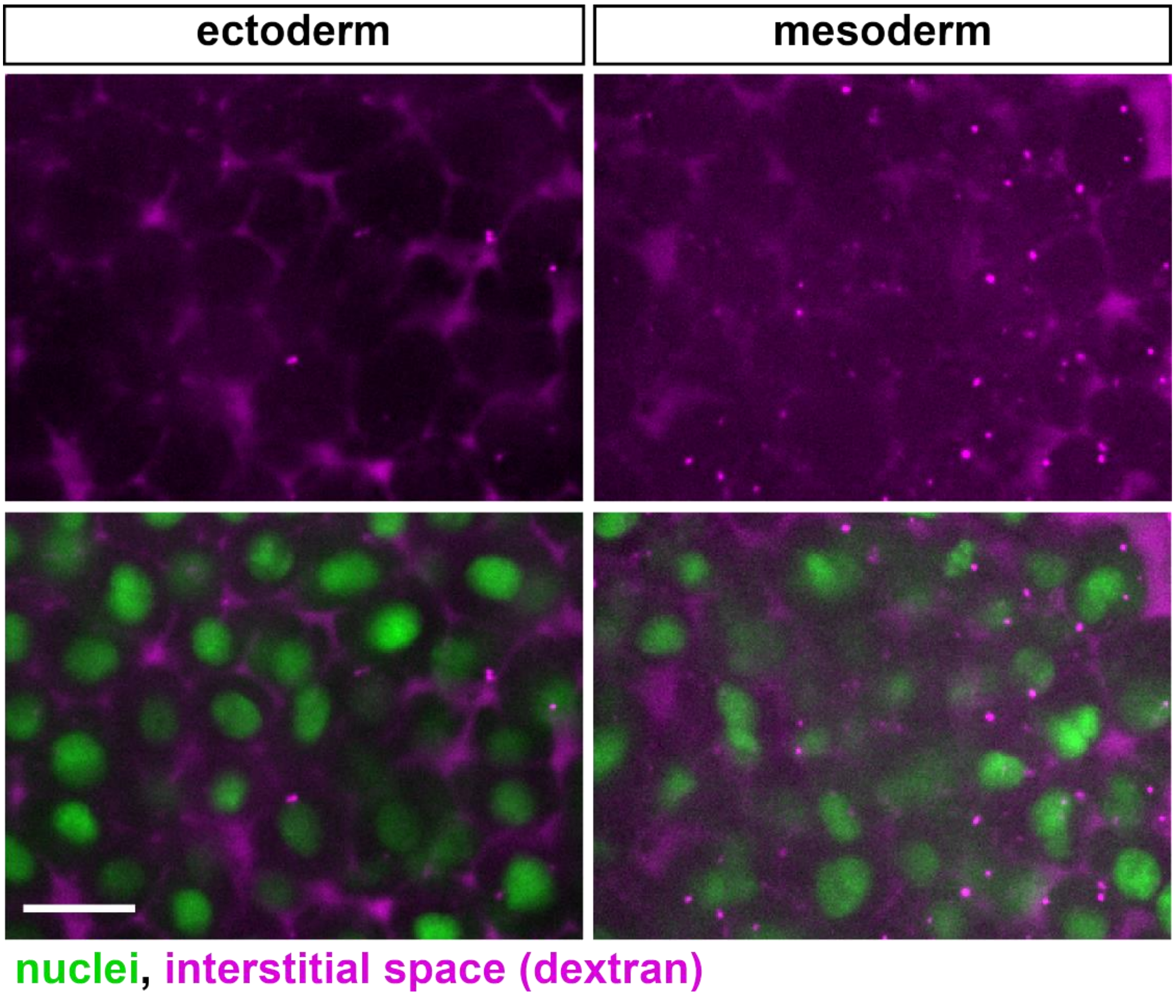

Figure R1. Interstitial space in converted embryos. Example images of ectoderm- and mesoderm-converted embryos. The embryos were injected at 4 hpf with 1 nl of 0.5 mg/ml Alexa-Fluor 680-conjugated dextran (10000 MW) into the interstitial space and imaged at 8 hpf. Upper panels present images of labeled dextran (magenta), while in lower panels these images are merged with the channel of H2A-tagBFP labeled nuclei (green). Scale bar, 20 µm.

© 2023. Published by The Company of Biologists under the terms of the Creative Commons Attribution License (https://creativecommons.org/licenses/by/4.0/).

For a paper that focusses on the impact of tissue environment there is little investigation into the respective tissue environments - almost all of the data show labelled PGCs on a black background.

Our imaging indeed focused on the dynamic behavior of the PGCs. We show in new Fig S1B a co-labelling of the PGCs and cells belonging to the two germ layers.

3. Excluding (or integrating) the previously described role for cadherins in regulating PGC blebbing

Previous work by the Raz lab has shown that differences in e-cadherin activity in surrounding tissues can influence PGC blebbing (albeit measured as a change in polarity). Given that ectoderm-only and mesoderm-only embryos will show differences in e-cad activity, this reviewer is surprised that this alternative interpretation was not explored here, or even mentioned in the discussion. While the bulk expression of e-cad may not vary detectably, due to the known maternal expression, it is highly likely that these germ layers will differ in e-cad activity, due to differences in stability, presentation etc. Moreover, it is predictable that mesoderm-only embryos will have n-cad activity which could also have an effect via a cadherin-based polarization mechanism.

Recommendation: The authors should rule out that the differences in PGC behavior in different tissues are not due to the e-cadherin based polarisation mechanism that they've previously described. If there are already compelling arguments against these should be covered in the discussion (the Grimaldi paper is currently cited as a source of methods but the biological findings of this work are important to discuss here).

In Grimaldi et al., we considered the main role of E-cadherin in PGC migration to impede retrograde actin flow, thereby helping to focus blebbing at the cell front. Lack of E-cadherin in environment manifests in PGCs by increased bleb frequency and enhanced retrograde actin flow, which results in reduced track straightness (Grimaldi et al., 2020). Measurements of E-cadherin protein levels conducted in response to this comment (new Fig. S6) indicate that ectoderm-converted embryos express lower amounts of E-cadherin than mesoderm-converted embryos do, making it theoretically possible that PGCs are affected by this parameter in the same manner. However, the difference in the level of E-cadherin between the two germ layers is not that extreme as in Grimaldi et al. Indeed, PGCs migrating within ectodermal cells, where E-cadherin level is lower than that in the mesoderm, form fewer blebs, with no difference in retrograde flow, nor in track straightness. Together, we concluded that the relatively small differences in E-cadherin expression between the mesoderm and ectoderm do not reach a level that differentially affects the migration. This issue is now presented and discussed in lines 190-202 and the Grimaldi et al paper is now mentioned in the main text.

Minor

4. Figure 4 investigates how differences in PGC 'self-organized polarity' could explain the described differences in bleb frequency. Using live reporters, they show that both RhoA activity is increased and erzin localization is expanded towards the cell front in ectodermal environments, making these cells less likely to bleb. The model they present - where a 'moderate', not high, increase in RhoA activity increases cortical coupling and thus prevents blebs - is a nice extension of that first presented in their Olguin-Olguin paper. However, here the new idea is not tested experimentally and is thus less convincing than the previous study.

To provide further support for the model according to which mild upregulation in RhoA activity reduces blebbing, we investigated the contractility more directly by measuring MLC-FRET and indeed observed no change in this parameter (new Fig. 3C, lines 144-147 in the main text). In addition, to test experimentally the effect of RhoA on ezrin distribution, we mildly increased the activity of RhoA in PGCs that reside in mesodermal tissue. This resulted in ezrin distribution similar to that observed in PGCs that reside in ectoderm-converted tissue. The results of these experiments show that a mild increase in Rho activity can broaden the distribution of ERM, supporting the proposed model. These results are presented in new Fig. 3H-I, and in lines 156-158 in the main text.

© 2023. Published by The Company of Biologists under the terms of the Creative Commons Attribution License (https://creativecommons.org/licenses/by/4.0/).

Reviewer 2 Advance Summary and Potential Significance to Field:
Truszkowski et al. explore how migrating Zebrafish primordial germ cells (PGCs) acutely respond to and adapt to cellular environments with different physical properties. The motivations for this study are twofold- (1) Zebrafish PGCs are born in distinct locations and may need to migrate within different cellular contexts with potentially different properties, and (2) previous work in the Raz lab indicated that PGC motility is obstructed by different barriers during developmental migration, such as the gut and notochord. To decouple whether the acute response to barriers (Loss of polarity) is due to mechano-sensing vs signaling, they inject hydrogels into the embryo and observe how non-directionally migrating PGCs respond. These experiments are followed up by observing PGCs in ectopically transformed embryos that are all ectoderm or mesoderm, with the ectoderm previously shown to have higher cortical tension. PGCs can surprisingly migrate with a similar speed and persistence in either cellular environment due to an increase in actin-based pseudopod motility at the expense of bleb-based motility in the ectoderm. The adaptive mechanism put forth is that PGCs increase RhoA activity globally and distribute ezrin more evenly across the membrane as to restrict bleb formation.
The conclusions and experiments are generally sound but require further clarification with additional data/revisions to the text.

We thank the reviewer for the comments and suggestions. We hope that the changes in the text and additional results improve the clarity of the paper.

Reviewer 2 Comments for the Author:
Comments-
1. Line 93- The authors state that the hydrogels utilized (Fig. S1) have physical properties measured as young's modulus which are within the physiological range that a PGC might encounter. However, there is no discussion of the associated physical properties (i.e. young's modulus measurement) of the embryonic cells that PGCs normally migrate within. For the previously described barriers that cause a repolarization of PGC migration (gut, notochord), is the stiffer gel within the range of these structures?

Our measurements suggest that the stiffness of the soft gel (500 Pa) is within the range of zebrafish embryonic tissues at similar stages of development (170 Pa – Tomizawa et al., 2019). The stiffness of the stiff gel (4.1 kPa) are similar to those of stromal connective tissues in adult organisms (Cox and Erlen, 2011). We do not know what the stiffnesses of the developing notochord and the enveloping ECM around it is, nor that of the developing gut.
To present the gel experiments more carefully, we removed the comparison to the embryonic tissues and just describe the experiments. Thus, in the revised version of the paper, we introduce the gels as a way to dissect the ability to perceive and respond to different levels of stiffness from responses to other cues (lines 203-211 in the main text).

2. In the hydrogel experiments, the PGCs, at least from the included movies and still images, primarily use actin-rich protrusions rather than the blebs one typically associates with these PGCs. This begs the question, how do PGC blebs interact with these hydrogels and if the apparent stiffness sensing mechanism is dependent upon a branched actin network present in the pseudopod.

To address this issue, we investigated the type of protrusion produced by the cells at the time of interaction with the gels. Interestingly, the cells reacted primarily when actin-rich protrusions were involved. This observation suggests that sensing of gel stiffness and the reactions involve effects on polymerized actin at the cell front. This point is now presented and discussed in lines 218-222 of the main text.

3. Are PGC migration speed and track straightness similar when migrating within control and dominant negative ROCK expressing ectoderm? If one assumes the cell surface molecule repertoire remains similar, this data could provide evidence that PGCs are adapting to the physical properties of the ectoderm rather than responding to surface molecules on the ectoderm vs mesoderm. The ectoderm and mesoderm express different levels of E-cadherin and this group has noted changes in PGCs migration in response to environmental cadherin levels. Can changes in E-cadherin levels on PGCs or environment alter morphology?

© 2023. Published by The Company of Biologists under the terms of the Creative Commons Attribution License (https://creativecommons.org/licenses/by/4.0/).

To determine if the change in blebbing frequency we observe in transplanted PGCs is part of their adaptation to physical properties of the environment, we transplanted PGCs into ectodermal environments expressing control or dominant-negative ROCK. In both control and DN-ROCK ectodermal environments, PGCs exhibited similar speed and track straightness (This result is presented in new figure, Fig. S4, and lines 111-114 in main text), consistent with the idea that changes in bleb frequency in response to the altered cortical tension of the environment lead to maintenance of migration speed.
To examine the effect of cadherin levels on PGC behavior, we compared changes in PGC behavior in E-cadherin depleted environment published in Grimaldi et al., 2020 with the behavior in the two germ layers. In an environment strongly depleted of E-cadherin PGCs form more blebs, while exhibiting faster retrograde actin flow and reduced track straightness (Grimaldi et al., 2020) (a finding we reproduced and do not show). We show that within the ectodermal environment PGCs produce fewer blebs as compared with their activity in mesodermal tissue in which E-cadherin level is relatively higher (an opposite trend from that shown in Grimaldi et al., 2020), while retrograde actin flow and track straightness are not different. From these results we conclude that the difference in E-cadherin expression between the two tissues are not responsible for the changes in cell behavior that we observe. We discuss these issues in lines 190-202 of the revised version of the manuscript.

4. Along these lines, it would be interesting to see if bleb size and frequency also decrease when PGCs are migrating within mesoderm with increased cortical tension through expression of constitutively active ROCK etc. This would show that this adaptive mechanism is more general and not specific to ectoderm.

In response to this comment, we transplanted PGCs into mesoderm-converted embryos in which we manipulated cortex tension by overexpressing a constitutively-active version of MLCK. This treatment led to a reduction in bleb formation frequency in PGCs (new Fig 2G-I, lines 105-107 in the main text). This finding is in line with the results obtained in ectodermal context, where lowering cortex tension resulted in an increase in bleb frequency.

5. Although identifying the causative upstream mechanism in which PGCs adapt to increased compression from the cellular environment is beyond the scope of this work, it would be interesting to see if PGCs which are "locked" into a particular migration mode fail to adapt when migrating within all ectoderm vs mesoderm. For example, in previous work from this group, PGCs overexpressing MLCK extend elongated protrusions and are still able to migrate.

To address this question, we increased the number of blebs migrating PGC form by expressing in the PGCs low levels of a dominant-negative form of Rac1(DN-Rac1). This treatment reproducibly led to increased blebbing, while maintaining cell polarity (new Fig. 4A, presumably by reducing the level of actin at the cortex). Interestingly, this manipulation led to a more pronounced reduction in PGC migration speed in ectodermal environment (47% vs. 25% in ectoderm vs mesoderm respectively, new Fig. 4B-C, lines 161-172). As suggested by the referee, this result shows that 'locking' PGCs in blebbing mode interferes with their migration speed more readily in ectodermal environment.

6. Please include representative FRET images for RhoA and Rac1 in Fig 4.

As requested, representative images are now provided in Fig. 3A-B.

Reviewer 3 Advance Summary and Potential Significance to Field:
In this paper, Truszkowski et al investigate zebrafish primordial germ cells (PGCs) migrating within different embryonic germ layers, and find that PGC migratory behavior is different between ectoderm and mesoderm environments. They show that PGCs in ectoderm produce smaller and less frequent blebs and more actin-rich protrusions than cells within mesodermal tissue. They also suggest a mechanism: PGCs within ectoderm tissue display elevated RhoA activity, which the authors propose leads to increased ERM activity and thus reduced blebbing.
Finally, the authors propose that differences in tissue mechanics between ectoderm and mesoderm cause the observed differences in PGC behavior.

© 2023. Published by The Company of Biologists under the terms of the Creative Commons Attribution License (https://creativecommons.org/licenses/by/4.0/).

The work is novel and potentially interesting. PGCs are a rare example of cells that migrate through changing environments in vivo in physiological conditions. They are thus an excellent system to study how the environment affects cell migration, and the findings presented here might have important impact in other systems where migrating cell face environmental changes, e.g. during cancer dissemination. However, some of the key conclusions are not, at this point, supported by the experiments, and several other points need to be addressed either through experiments or through clarifications.

We thank the reviewer for the constructive comments and hope that the additional experiments and clarifications sufficiently support the key conclusions.

Reviewer 3 Comments for the Author:
My main concern lies with the interpretation proposed by the authors that the differences in migratory behavior they observe are due to differences in mechanics between ectoderm and mesoderm. This conclusion is, at present, poorly supported by the data, and the discussion of mechanics is overall very confusing. Specifically:
1. The authors first test the effects of mechanics by introducing hydrogels of different stiffnesses into an embryo – Figure S1. However, the data presented in this figure are not fully convincing. Given the low numbers, the conclusion relies on a few cells displaying a “polarity loss” rather than “no change” upon contacting stiffer gels. But is 5 or 6 minutes long enough a movie for this analysis? Have the authors tried observing for a longer time? For instance, in the videos presented the cells approach the hydrogels at quite different angles, could it be that “no change” turns into “turn” or “polarity loss” if observed for longer? More fundamentally, the rationale of this experiment within the context of the paper is unclear: how does hydrogel stiffness relate to cortical tension of neighboring cells? Stiffness and tension are very different physical properties (see more on this below).

The title of the graph in question was perhaps misleading and we changed it (see Fig 4G). The movies we present are 5-6 minutes long for the following reason. PGCs lose their polarity periodically and re- establish it, in the context of this work in a random direction due to lack of guidance cues. Thus, if one acquires movies for long enough, loss of polarity will eventually be observed (‘tumbling behavior’, Reichman-Fried et al., 2004), regardless of contacting the gel. This point can be observed in Figure 4G for control cells i.e. cells that do not touch the gel. Therefore, we decided to restrict the analysis to 6 minutes and focus on the immediate response to the interaction with the gel. As shown in Figure 4G, cells interacting with soft gels behave similarly to cells that did not interact with gels. In contrast, cells that interacted with the stiff gels always reacted to the interaction by polarity loss or turn. In response to the comments of the referee, we explain this point better in the figure legend for Fig. 4E (lines 698-703), where the schematic of the experiment is presented.

Following the reviewer’s suggestion, we determined the time required for a PGCs to change their polarity (lost, or turn) following interaction with the gel. We found that when monitored for a long duration, all cells eventually change their polarity, but contacting the stiffer gel led to a faster change in polarity (Fig. S7). In addition, to increase the confidence in the results, we recorded additional cell interactions with the soft gel. In addition, we re-analyzed the previously acquired data, double blinding it reaching the conclusion that PGCs interacting with the stiff gels always changed their actin polarity within the time interval of 5 minutes (Fig 4G).
The rationale of the hydrogel experiments was to compare the reaction to structures that differ in only one physical parameter that we can control. In this setup, we could ensure that the only difference between the encountered structures is the stiffness – in these dextran gels the only difference is the concentration of the cross linkers, such that the porosity is the same. Indeed, from the perspective of the probed structure stiffness and cortical tension are different parameters (Salbreaux et al., 2012). The way we considered it, the probing cell (in this case, a PGC) does not distinguish between these parameters from the outside but rather recognizes the resistance to deformation. We refer to this point in the main text, hoping that it is clearer now (lines 206-211).

2. A directly related question is how do the authors propose that the cells achieve mechanosensing, and what do they actually sense? To my knowledge, current understanding

© 2023. Published by The Company of Biologists under the terms of the Creative Commons Attribution License (https://creativecommons.org/licenses/by/4.0/).

of substrate stiffness sensing is that it relies on focal adhesions. Do PGCs form focal adhesions? And if not, how do the authors propose they could sense hydrogel stiffness? If this is a focal adhesion independent stiffness sensing mechanism, that would be a very exciting finding that should be explicitly discussed, but there is at this point insufficient evidence that stiffness sensing takes place at all in this system.

We have no indication that PGCs form focal adhesions and the cells can migrate when integrin function is inhibited (Kardash et al., 2010).
In the same direction, the gels do not possess properties that would allow them to be specifically bound by the cell. We thus consider the gel experiments valuable in the sense of showing that the cells can respond to "inert" structures and discriminate between soft and stiff entities. We find that most of the responses occur only after actin-rich front (rather than blebs) comes into contact with the gel (also see response to point 2. of Reviewer 2). Therefore, we propose that sensing of gel stiffness is mediated by the effect of the gel on the advance of the actin-rich protrusion. We present these results and the possible implications in the main text of the revised paper. A related finding in an in vitro setting was recently published by the Sixt group (Gaertner et al., 2022) and we cite this work (lines 218-222).

3. For the cells migrating in mesoderm and ectoderm: the authors write several times that these two tissues exhibit different biophysical properties, referring to Krieg's 2008 paper. However, to my knowledge, that paper measured the cortical tension of individual cells isolated from ectoderm- and mesoderm-converted embryos. It is not clear at all how the mechanical properties of single isolated cells translates to the mechanical properties of the tissue. The authors refer to tissue tension but also sometimes to cell tension within the tissue. Tissue tension is likely more determined by the ECM than by the mechanics of individual cells. The authors need to clarify that, as I presume, they mean to refer to the mechanics of the individual cells, not of the tissue (or provide some references on actual tissue mechanics).

The referee is right and we indeed meant to refer to the interaction of the single-migrating cells (the PGCs) with cells, rather than with the whole tissue. We corrected the text accordingly and made it clear that the parameter the cells respond to is the tension of individual cells in the environment.

4. The authors also occasionally use the word stiffness, apparently as a synonym to tension (e.g. l. 140). Stiffness is a very different physical property to cortical tension, do the authors actually mean stiffness in these instances? It is plausible that cortical tension could be sensed in a similar manner to stiffness, however in principle, tension does not directly correlate with stiffness.
Assuming they mean tension, as this is what has been measured in Krieg et al 2008, it would be interesting to speculate how they propose cells might sense cortical tension. Some previous work by the authors reported that PGCs form cadherin bonds to neighboring cells, is this where they propose cortex tension would be sensed?

In the case of cell-cell interaction we indeed meant to refer to cortical tension, and we changed the text accordingly. The term "stiffness" referred to the hydrogel, where we considered stiffness to be a property that could resemble cortical tension, from the perspective of the probing cell (see also on the reply to point 1. of Reviewer 3). We would appreciate further advice regarding this issue to ensure that the wording is correct.
As pointed by the reviewer, E-cadherin could in principle play a role in sensing cortical tension. We cannot exclude the role of E-cadherin in sensing in the context of cell behavior in the different environments, but the results of the hydrogel experiments show that sensing can take place also independently of E-cadherin-mediated adhesion. Thus, sensing of purely physical features could in principle dictate cell behavior in embryos. We discuss this in the text of the revised paper in 213-217.

5. The evidence that the differences in PGC behavior are actually due to differences in cell mechanics in the different tissues is rather minimal, the conclusion appears to rely on the observed increase in blebbing frequency (but not size) in dn rock ectoderm hosts. This

© 2023. Published by The Company of Biologists under the terms of the Creative Commons Attribution License (https://creativecommons.org/licenses/by/4.0/).

is very correlative and the authors need to either substantiate the experimental support for their conclusion (e.g. by investigating how the mechanosensing is achieved), or strongly tone down their conclusions.

In an attempt to increase the confidence in the idea that cortical tension affects blebbing frequency, we conducted an additional experiment where non-manipulated PGCs were transplanted into mesodermal cellular environment expressing the constitutively active MLCK (new results presented in Fig. 2G-I). We observed reduced bleb frequency in the transplanted cells, consistent with the previous observation that lowering cortical tension results in increased bleb frequency (Fig. 2D-F in the revised version of the report). Interestingly, in both manipulations PGC bleb size was not affected, suggesting that other parameters influence different features of blebbing. In response to the request of the referee, this conclusion is now toned down in the abstract (line 34-35) and main text (lines 67-68 and 107-111).

Overall, unless further data more clearly supporting a role for mechanics can be provided, I would suggest considerably toning down the statements that the differences in behavior observed are due to differences in mechanics and either removing the hydrogel experiment or moving it to the end of the paper, as a speculation point rather than a starting point.

To address this point, we present the hydrogel experiments only at the end of manuscript and present the conclusions as “suggestive” (lines 215-217). The results of the experiments are referred to in the abstract without any interpretation (lines 38-40).

Minor comments:

1. In the introduction, the authors present PGCs as “an accessible in vivo model” for how cell migration adapts to the environment. Do PGCs actually move between ectoderm to mesoderm in vivo (the authors suggest they do, but only rather late in the paper, l. 160, maybe this should be discussed earlier / more extensively?), and if so, is it possible to assess if they display the same changes in protrusion formation?

In wild-type embryos we occasionally observe cells in the ectoderm and the cells should leave this tissue to reach the mesoderm, where their target is located. Also, we occasionally observe PGCs that exhibit a bias towards formation more actin-rich protrusions, in particular when the chemokine receptor or ligand were knocked down (as in embryos from Fig S1A). The actual transition between the germ layers is not frequent or predicted, such that we could describe it to a level done for the converted embryos.

2. “cells can alter their protrusion type or migration mode when located in different 2D and 3D environments” (72-73): this should reference Liu’s 2015 paper (which is cited later in the manuscript), which reports an extensive study of the behavior of numerous cell types in confinement.

Liu et al., 2015 is now cited in the mentioned line (now 62).

3. In the mutant embryo, the authors observe PGCs within all 3 germ layers: ectoderm, mesoderm, and endoderm. Yet there is no further discussion of PGCs in the endoderm. Could the authors clarify why they focused on the ectoderm and mesoderm for the rest of the study?

Conversion of the whole embryo into endodermal tissue is possible (Krieg et al., 2008). Indeed, at early stages of the project, this path was followed as well.
However, as done for the other experimental setups, to ensure that that the germ cells themselves are not altered by the treatment, we examined the expression of germ cell markers within them. When forcing the embryonic cells to differentiate into endoderm, we noted a slight change in the expression level of *nanos* and *vasa* RNA in the PGCs, which could potentially influence results in some of the experiments. To ensure that the differences we detect indeed result solely from differences in the environment, we focused on the ectoderm and mesoderm treatments that did not affect gene expression in the PGCs. The differences in behavior were

© 2023. Published by The Company of Biologists under the terms of the Creative Commons Attribution License (https://creativecommons.org/licenses/by/4.0/).

observed also in untreated PGCs that were transplanted into single germ layer embryos, such that changes within the PGCs were completely ruled out.

4. In the single germ layer embryos, are cells integrated within the tissue or is it possible they lie on the tissue boundary (i.e. between tissue and yolk)? A figure in the style of S2A for the converted embryos would be useful.

As suggested by the reviewer, we now include an example of PGCs integrated within the tissue of converted embryos (new Fig. S1B).

5. "These results [that PGCs in ectoderm form fewer/smaller blebs] were further supported by analyzing blebbing in wild-type PGCs transplanted into either ectoderm-converted or control embryos". Why was this not done in the mesoderm-converted embryo also?

In the originally submitted report we conducted this rather challenging experiment only for the ectodermal environment, as the differences were striking and we wanted to confirm them in another way. In the current version of the manuscript we also describe transplantations into mesoderm- converted embryos in the context of manipulating cell cortex tension.

6. From the methods, it appears that many of the quantifications were done manually (e.g. Figure 2 bleb frequency and size, Figure S1 behavior quantifications). This is not a problem per se, however, given the small numbers, such manual quantifications should be done double- blinded. Was this the case?

Allocation of embryos into respective groups was random, but the original analysis was not done blinded. To increase the confidence in hydrogel experiments, we now double blinded the analysis and present the results of this new analysis in Figures 4E-G and S7. This statement is now included in the Materials and methods section (lines 456-459).

7. Could the authors show a 3-dimensional rendering of the protrusions shown in Figure 3B? Are these flat lamellipodia-like protrusions or 3D shaped? And if they are not flat, is it entirely clear they are not some form of blebbing? Movie 4 does, at first glance, appear to show some bleb-like activity, at least from the 2D projection shown.

Based on our observations, the protrusions formed by PGCs in ectodermal environment are three dimensional. We now provide a movie with orthogonal projection that encompass the entire protrusion in Z-plane (Movie 3). In this movie, one can compare the actin-rich protrusion of polarized cell (left cell) with the blebs generated by the unpolarized, tumbling cell (right).

8. Examples of FRET images quantified in Figure 4A-C should be provided.

As requested, representative FRET images are now shown (Fig 3A-B in the revised manuscript).

9. Figure 4C shows a much wider distribution in the RhoA front/back ratio of PGCs in ectoderm compared to mesoderm. Is this meaningful? Also, could the authors provide the actual p-values, rather than "ns"?

As requested by the reviewer, P-values are now provided in all the graphs.
We observed this difference in distribution as well, a point that might be related to other differences in migration that we did not assay for. We did not feel we could draw specific conclusions from this observation.

10. Figure S2A is a projection of a 3D stack, and therefore does show magenta specks within the PGC (especially center - mesoderm - image). Since the purpose of this figure is to demonstrate the mutual exclusivity of germ layer markers and PGC markers, is there another way to represent this that would be clearer?

© 2023. Published by The Company of Biologists under the terms of the Creative Commons Attribution License (https://creativecommons.org/licenses/by/4.0/).

To address this issue, we repeated the experiment using a scanning confocal instead of a spinning disk microscope. In this way, we excluded germ-layer specific specks from the PGCs. The results are presented now in new Fig. S1A.

References:

Cox, T. R. and Erler, J. T. (2011). Remodeling and homeostasis of the extracellular matrix: implications for fibrotic diseases and cancer. *Dis. Model. Mech.* **4**, 165–178.

Gaertner, F., Reis-Rodrigues, P., de Vries, I., Hons, M., Aguilera, J., Riedl, M., Leithner, A., Tasciyan, S., Kopf, A., Merrin, J., et al. (2022). WASp triggers mechanosensitive actin patches to facilitate immune cell migration in dense tissues. *Dev. Cell* **57**, 47-62.e9.

Grimaldi, C., Schumacher, I., Boquet-Pujadas, A., Tarbashevich, K., Vos, B. E., Bandemer, J., Schick, J., Aalto, A., Olivo-Marin, J.-C., Betz, T., et al. (2020). E-cadherin focuses protrusion formation at the front of migrating cells by impeding actin flow. *Nat. Commun.* **11**, 5397.

Krens, S. F. G., Veldhuis, J. H., Barone, V., Čapek, D., Maître, J.-L., Brodland, G. W. and Heisenberg, C.-P. (2017). Interstitial fluid osmolarity modulates the action of differential tissue surface tension in progenitor cell segregation during gastrulation. *Development* **144**, 1798–1806.

Krieg, M., Arboleda-Estudillo, Y., Puech, P.-H., Käfer, J., Graner, F., Müller, D. J. and Heisenberg, C.-P. (2008). Tensile forces govern germ-layer organization in zebrafish. *Nat. Cell Biol.* **10**, 429–436.

Salbreux, G., Charras, G. and Paluch, E. (2012). Actin cortex mechanics and cellular morphogenesis. *Trends Cell Biol.* **22**, 536–545.

Tomizawa, Y., Dixit, K., Daggett, D. and Hoshino, K. (2019). Biocompatible Cantilevers for Mechanical Characterization of Zebrafish Embryos using Image Analysis. *Sensors* **19**, 1506.

Second decision letter

MS ID#: DEVELOP/2022/200603

MS TITLE: Primordial germ cells adjust their protrusion type while migrating in different tissue contexts in vivo

AUTHORS: Lukasz Truszkowski, Dilek Batur, Hongyan Long, Katsiaryna Tarbashevich, Bart E Vos, Britta Trappmann, and Erez Raz
ARTICLE TYPE: Research Report

I am happy to tell you that your manuscript has been accepted for publication in Development, pending our standard ethics checks.

Reviewer 1

Advance summary and potential significance to field

The manuscript by Truszkowski and colleagues addresses the important and timely topic of how migrating cell behaviours emerge from interactions between the cells and their tissue environment. The experiments presented here are ingenious, wide-ranging and are likely to inspire future studies. This reviewer considers the results, and discussion thereof, interesting and useful for the readership of Development.

© 2023. Published by The Company of Biologists under the terms of the Creative Commons Attribution License (https://creativecommons.org/licenses/by/4.0/).

Comments for the author

The authors have done a good job of revising their manuscript, which is strengthened as a result. I have no further comments, other than to congratulate the authors on their interesting study.

Reviewer 2

Advance summary and potential significance to field

Truszkowski et al. explore how migrating Zebrafish primordial germ cells (PGCs) acutely respond to and adapt to cellular environments with different physical properties. The motivations for this study are twofold- (1) Zebrafish PGCs are born in distinct locations and may need to migrate within different cellular contexts with potentially different properties, and (2) previous work in the Raz lab indicated that PGC motility is obstructed by different barriers during developmental migration, such as the gut and notochord. To decouple whether the acute response to barriers (Loss of polarity) is due to mechano-sensing vs signaling, they inject hydrogels into the embryo and observe how non-directionally migrating PGCs respond. These experiments are followed up by observing PGCs in ectopically transformed embryos that are all ectoderm or mesoderm, with the ectoderm previously shown to have higher cortical tension. PGCs can surprisingly migrate with a similar speed and persistence in either cellular environment due to an increase in actin-based pseudopod motility at the expense of bleb-based motility in the ectoderm. The adaptive mechanism put forth is that PGCs increase RhoA activity globally and distribute ezrin more evenly across the membrane as to restrict bleb formation. The conclusions and experiments are generally sound. The authors provided clarification and additional experiments that addressed our questions and suggestions.

Comments for the author

no further revisions are required.

© 2023. Published by The Company of Biologists under the terms of the Creative Commons Attribution License (https://creativecommons.org/licenses/by/4.0/).
